# Nickel-Induced Lattice Defects Limit Proton Uptake in Barium Zirconate Electrolytes

**DOI:** 10.1021/jacs.5c13935

**Published:** 2025-12-12

**Authors:** Yabing Wen, Andreas Rosnes, Bo Jiang, Øystein Prytz, Truls Norby, Reidar Haugsrud, Jonathan M. Polfus

**Affiliations:** † Department of Chemistry, Centre for Materials Science and Nanotechnology, 6305 University of Oslo, PO Box 1033, Blindern, NO-0315 Oslo, Norway; ‡ Department of Physics, Centre for Materials Science and Nanotechnology, 6305 University of Oslo, PO Box 1048, Blindern, NO-0316 Oslo, Norway

## Abstract

Nickel provides essential catalytic properties for hydrogen electrodes in proton-conducting ceramic electrochemical cells. However, Ni diminishes the hydration capability and proton conductivity when incorporated into electrolyte materials including BaZr_0.8_Yb_0.2_O_3−δ_ studied here. Through semiquantitative atomic-resolution scanning transmission electron microscopy, density functional theory simulations, X-ray total scattering, and absorption spectroscopy, we reveal that Ni forms point defect clusters with the Yb acceptors wherein oxygen vacancies are trapped and resist hydration. The resulting effective acceptor concentration is described by point defect reactions in quantitative agreement with thermogravimetric measurements of hydration for samples substituted with 2–5 mol % Ni by BaNiO_2_ addition. Moreover, excess B-site cations due to NiO addition induce the formation of antiphase boundaries (APBs) that are enriched in Yb and thereby deplete the bulk of acceptors, further suppressing hydration. The adverse effects of Ni are thereby resolved into two novel mechanisms, opening new avenues in point defect engineering for high-performance electrolytes.

## Graphical abstract

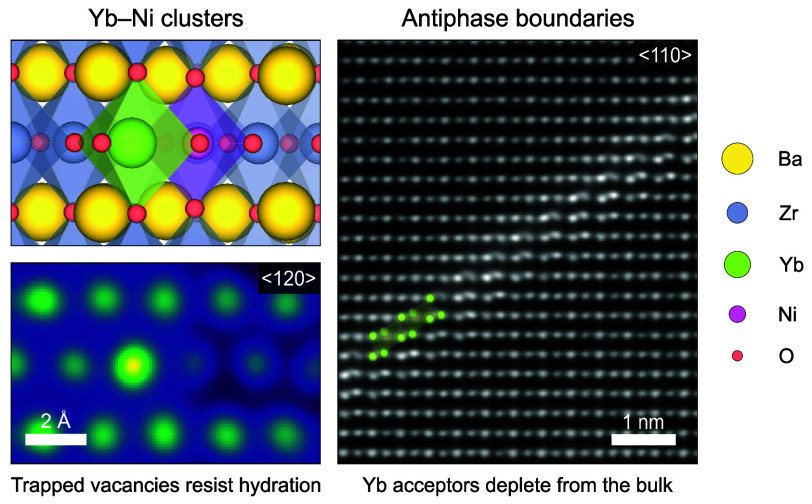

## Introduction

Solid solutions of barium zirconate and cerate, Ba­(Zr,Ce)­O_3−δ_, substituted with trivalent acceptors on the perovskite B-site have emerged as the leading proton-conducting electrolyte for electrochemical cells operating at intermediate temperatures (400–700 °C).^1–4^ Nickel is well-established as hydrogen electrocatalyst in composites with the electrolyte phase, wherein the electrode porosity is obtained upon in situ reduction of NiO to Ni.^5–7^ Co-sintering of the composite electrode and the electrolyte causes Ni to diffuse into the electrolyte, where it acts as a sintering aid crucial for obtaining dense and large grained electrolyte layers.^8–10^ Notably, the BaZrO_3_-based electrolyte can thereby be densified at approximately 1500 °C despite its refractory nature.^9,11^ However, Ni adversely impacts the effective acceptor concentration of the electrolyte, leading to reduced proton concentrations and conductivities.^12–14^ This may account for the scarce application of Ce-free BaZrO_3_-based electrolytes in proton-conducting ceramic cells although they exhibit superior bulk protonic conductivity and chemical stability.^15,16^

Formation of the secondary phase BaY_2_NiO_5_ corresponds to depletion of Y acceptors from the perovskite phase, accounting for the diminished saturation limit for hydration in BaZr_0.8_Y_0.2_O_3−δ_ with NiO addition.^17^ However, these secondary phases form only for compositions substituted with acceptors having a radius equal to or larger than Y^3+^, while smaller acceptors such as Sc, In, and Yb remain within the solubility limit.^18,19^ Yet, NiO retains its detrimental impact on hydration even in single-phase materials such as BaZr_0.8_Yb_0.2_O_3−δ_ and BaZr_0.4_Ce_0.4_Y_0.1_Yb_0.1_O_3−δ_.^9,20^ Nevertheless, Ni is confirmed by atomic resolution energy dispersive spectroscopy (EDS) within scanning transmission electron microscopy (STEM) to substitute the B-site, meaning that Ni acts as an acceptor that in principle should lead to increased proton uptake.^20^

The presence of excess B-site cations in perovskite oxides can lead to the formation of antiphase boundaries (APBs), which accommodate the cation nonstoichiometry.^21,22^ The addition of NiO to acceptor substituted BaZrO_3_ represents analogous B-site excess stoichiometries. However, the formation of APBs and their potential role in the hydration characteristics of electrolyte materials remains unexplored. Furthermore, APBs can act as fast cation diffusion pathways,^21^ and their formation in BaZrO_3_ may facilitate further Ni diffusion into the electrolyte during cosintering with the composite electrode.

To clarify the multifaceted role of Ni in single-phase proton-conducting perovskites, the hydration behavior is here systematically studied for BaZr_0.8_Yb_0.2_O_2.9_ (BZYb20) with additions of 2–5 mol % equimolar amounts of BaCO_3_ and NiO (denoted BaNiO_2_) or NiO, prepared by solid-state reactive sintering at 1500 °C. By combining direct STEM observations, X-ray total scattering and absorption spectroscopy, and density functional theory (DFT) simulations, we reveal that incorporation of Ni leads to point defect clustering within the bulk lattice that suppresses hydration of oxygen vacancies and thereby limits proton uptake. Furthermore, Yb-enriched APBs, formed exclusively in the B-site-excess system due to NiO addition, constitute another key mechanism that suppresses the hydration saturation limit.

## Results

### Structure and Proton Uptake

In studies of the effective acceptor concentration, it is crucial to account for any formation of impurity phases that may deplete the electrolyte of the acceptors. Synchrotron X-ray diffraction confirms the phase purity of BZYb20 with 5 mol % BaNiO_2_ (BZYb20-5BaNiO_2_) and 5 mol % NiO (BZYb20-5NiO) as shown in Figure 1a, and the phase purity of all samples are confirmed in Figure S1. X-ray pair distribution function (PDF) analysis of the local structure suggests a high degree of similarity with minor differences in the A–B and B–B distances between the two samples (Figure 1b, and more details are given in Figure S3). The oxidation states of Ni in BZYb20-5BaNiO_2_ and BZYb20-5NiO were investigated using the X-ray absorption near-edge structure (XANES) to elucidate the potential role of Ni as an acceptor. From the first derivative maxima of the normalized spectra (Figure S4), the charge state of Ni was estimated to be approximately 2.7 and 2.5 (Figure 1d) and Ni is henceforth treated as a trivalent acceptor in alignment with the predominance of Ni^3+^ substituted on the B-site of Y-doped BaZrO_3_.^23^

**Figure 1:**
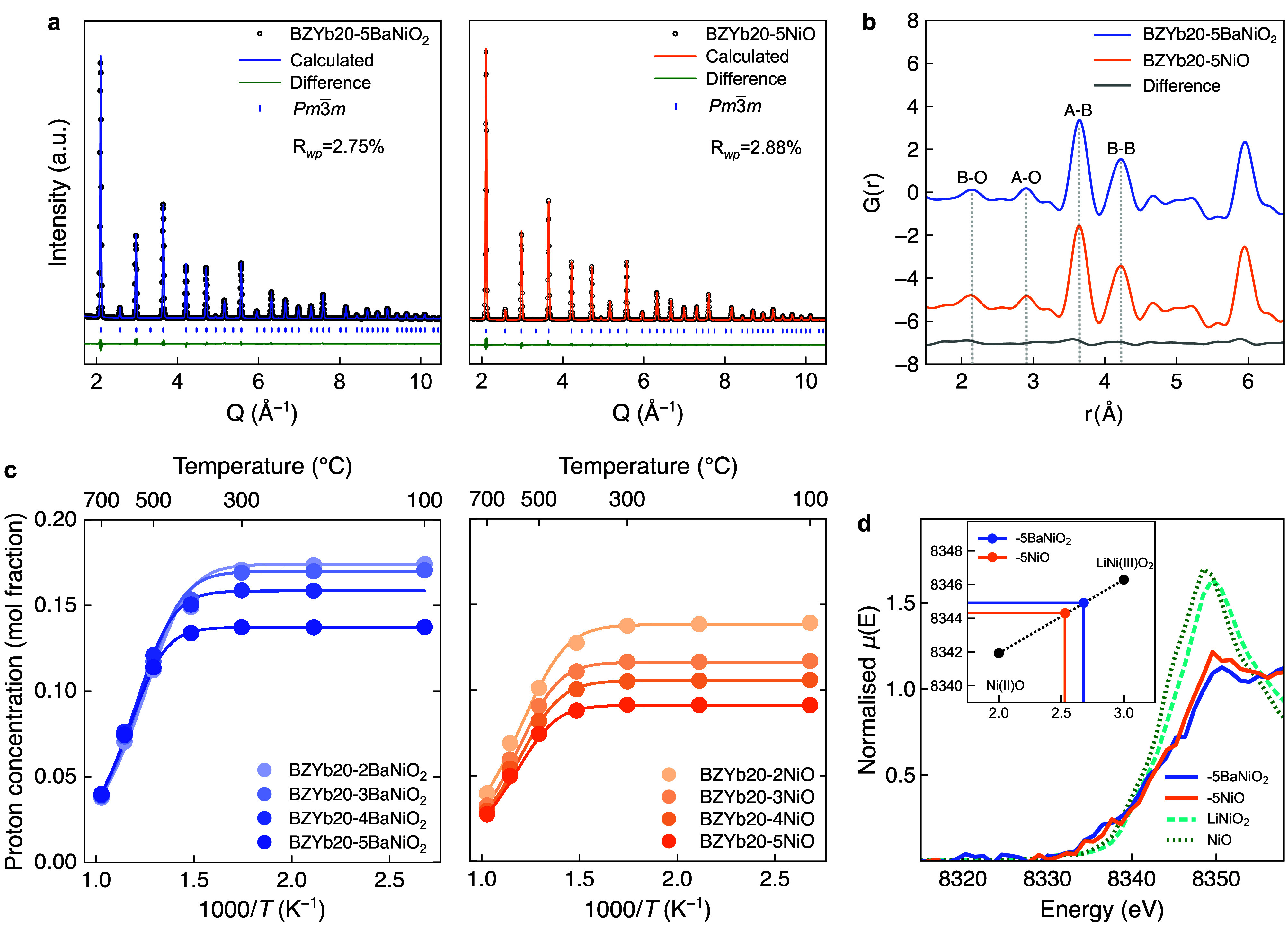
Structural characterization and hydration of BZYb20 with BaNiO_2_ and NiO. (a) Rietveld refinements of synchrotron X-ray diffraction patterns show a single cubic phase. (b) X-ray pair distribution functions for the short-range atomic correlations show the similarity in the local structure between samples with BaNiO_2_ and NiO addition. (c) Proton concentrations from thermogravimetry conducted in humidified N_2_ (*p*H_2_O = 0.02 atm). Solid lines are fitted by using thermodynamic parameters for the hydration reaction (Table S1) and a variable effective acceptor concentration. (d) XANES spectra including references for NiO and LiNiO_2_. Inset: Ni oxidation state as a function of the Ni K-edge position.

The saturation limit for proton uptake from water vapor, corresponding to the effective acceptor concentration, is quantified via thermogravimetric analysis of the hydration of oxygen vacancies, here written in Kröger-Vink notation:

$$H_{2}O\left(g\right)+v_{O}^{••}+O_{O}^{\times }⇌2{\mathrm{OH}}_{O}^{•}$$ 1

The measurements reveal that the hydration saturation limit decreases with an increasing amount of BaNiO_2_ or NiO compared to the nominal acceptor level of 20 mol % Yb (Figure 1c). Notably, the addition of BaNiO_2_ leads to less suppression of the hydration limit compared to NiO addition, consistent with trends in BaZr_0.8_Y_0.2_O_3−δ_.^24^

### Nanoscale Imaging of Point Defect Clusters

Intriguingly, the large differences in the atomic numbers of Ni (*Z* = 28) and Yb (*Z* = 70) relative to that of Zr (*Z* = 40) enable direct investigation of point defect clusters by STEM high-angle annular dark field (HAADF) imaging wherein columns enriched in Yb and Ni appear bright and dark, respectively. The ⟨120⟩ zone axis is utilized to distribute the B-site constituents over several atomic columns, thereby increasing the sensitivity to Ni and Yb occupancy, i.e., imaging at the lower-order ⟨110⟩ or ⟨100⟩ zone axes yields intensities closer to the B-site average. Under these conditions, the Ni containing samples exhibit inhomogeneous intensity distributions on the B-site (Figure 2a).

**Figure 2:**
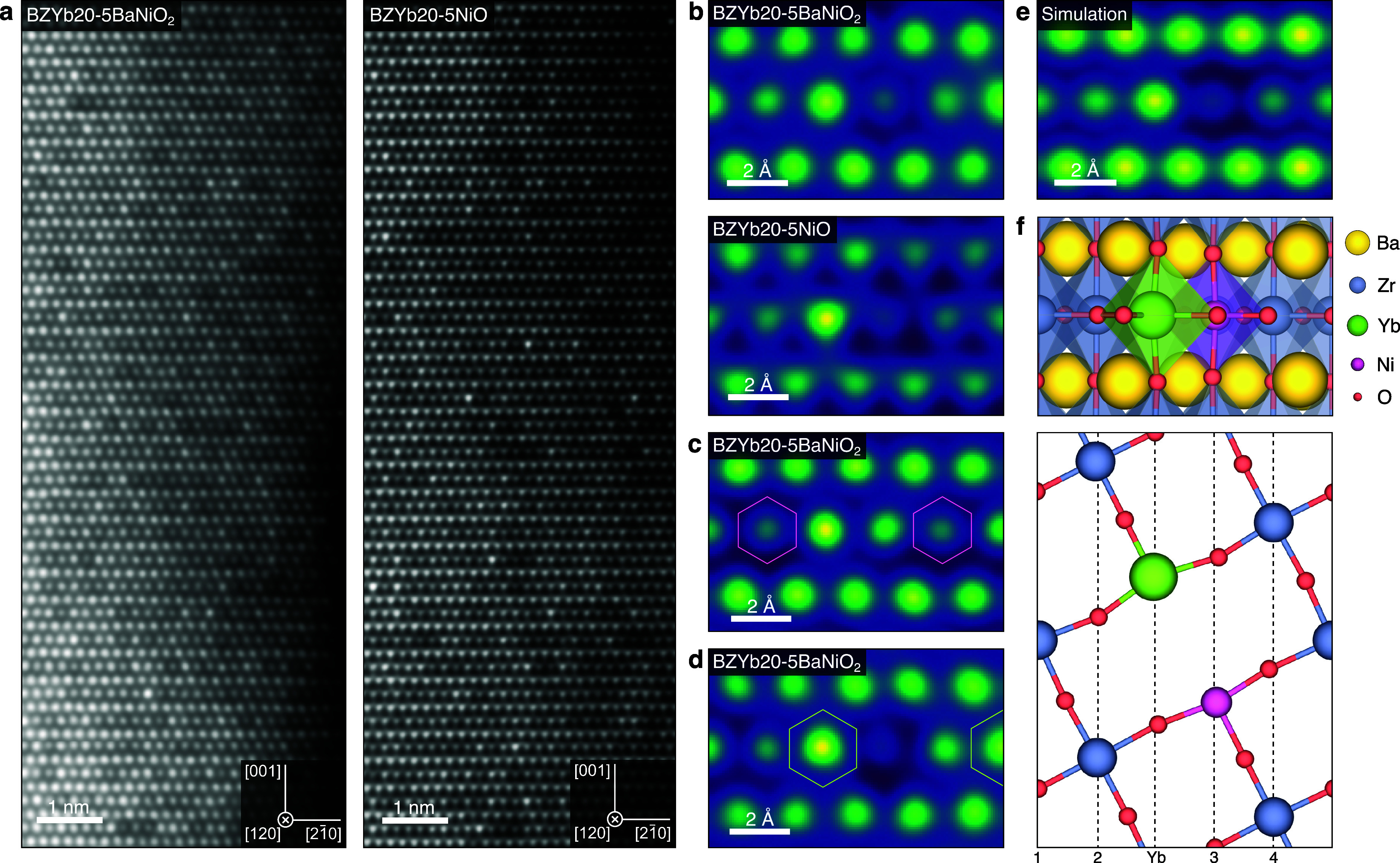
Nanoscale imaging of Yb–Ni point defect clusters. Experimental and simulated STEM-HAADF images at the ⟨120⟩ zone axis. (a) STEM-HAADF images of BZYb20-5BaNiO_2_ and BZYb20-5NiO showing inhomogeneous intensity distributions on the B-site. Magnified STEM-HAADF images in linear color scale of BZYb20-5BaNiO_2_ and BZYb20-5NiO with (b) Yb–Ni on neighboring columns, (c) Ni on nearest and second nearest neighbor columns (indicated by hexagons), and (d) Yb on two columns (indicated by hexagons) coordinated to a single Ni-enriched column. (e) Simulated HAADF image of Yb–Ni clusters with an occupancy of 0.5. (f) Visualization of a relaxed Yb–Ni cluster corresponding to the simulated image in (f) (top) and sideview of the ⟨120⟩ B-site columns with the Yb–Ni cluster in columns 3 and 4 (bottom). Yb is coordinated to B-sites at nearest neighbor columns 2 and 3 and second nearest neighbor columns 1 and 4.

Most importantly, columns enriched in Ni are observed adjacent to columns enriched in Yb in multiple locations (Figure S5), and the images correspond well with the simulated HAADF image of Yb–Ni clusters using the multislice method^25^ (Figure 2b,e). Here, another beneficial characteristic of the ⟨120⟩ zone axis is the lack of nearest neighbors within the B-site columns (Figure 2f), thereby facilitating the distribution of Yb–Ni clusters on neighboring columns. The B-site has nearest neighbors at two adjacent columns in each direction along [21̅0] (Figure 2f), meaning that Yb–Ni clusters can also appear on second nearest neighbor columns (Figure 2c). Additionally, the B-site has nearest neighbors in each direction along [001] (Figure S6). These features support that the inhomogeneous intensity distributions originate from Yb–Ni clusters with different crystallographic orientations.

Regions with pronounced intensity differences at two neighboring B-site columns are identified for semiquantitative analysis of the Yb and Ni occupancies. By comparison to simulated HAADF images with increasing occupancy of Yb–Ni clusters, i.e., equal Yb and Ni occupancy in neighboring columns, both integration of the column intensities by the Voronoi method (Figure 3a) and line profiles (Figure 3b) indicate Yb and Ni occupancies significantly higher than their nominal molar fractions, 0.2 and 0.05, respectively.

**Figure 3:**
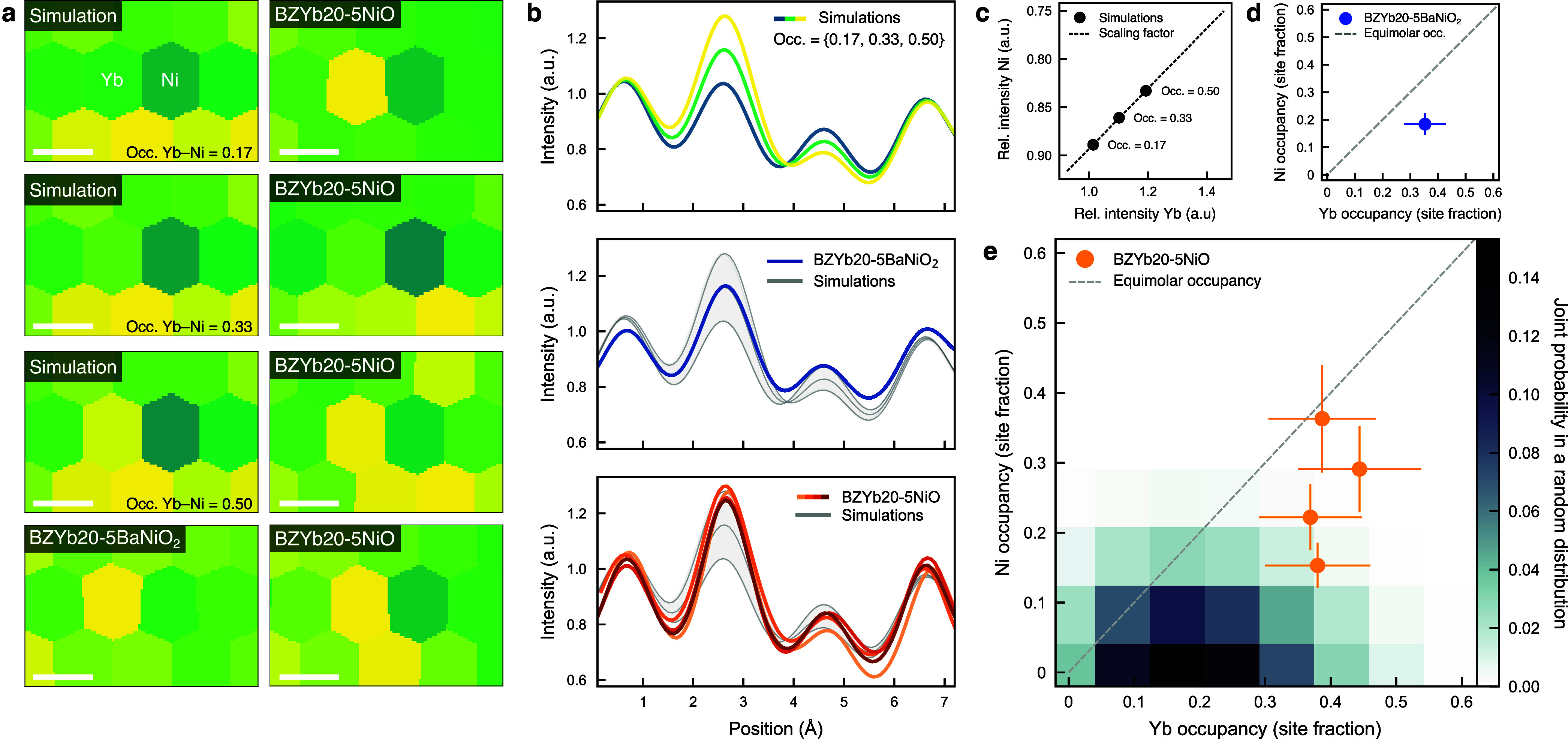
Semiquantitative STEM analysis of Yb–Ni clusters. (a, b) Voronoi maps and line profiles for simulations with increasing occupancy of Yb–Ni clusters and selected regions in BZYb20-5BaNiO_2_ and BZYb20-5NiO. (c) Scaling factor that relates relative intensity to occupancy obtained from a linear fit to the simulations. (d, e) Individual occupancies of Yb and Ni in neighboring columns. The error bars represent a sum of random and systematic errors with a main contribution from the detector nonlinearity. The overlaid map shows the joint probability of Yb and Ni occupancy in neighboring columns of 12 atoms for a random distribution with the highest probability for the nominal Yb and Ni molar fractions.

The individual occupancies of Yb and Ni can be estimated from the integrated column intensities relative to an internal reference, taken as the average of the B-site columns 2 and 4 (Figure 2f), and scaling them according to the simulations where the occupancies are known. The relative intensity differences from the simulations scale linearly to the individual occupancies of up to 0.5 (Figure 3c). The resulting occupancies are slightly higher for Yb compared to Ni (Figure 3d,e), consistent with the presence of additional nonclustered Yb in the Yb-rich columns. The analysis is facilitated by thin regions with fewer atoms in the columns, enhancing the sensitivity to Yb–Ni clusters while minimizing the influence of nonclustered Yb and clusters with different configurations that cause Yb and Ni to reside within the same column. Consequently, multiple suitable regions are identified in BZYb20-5NiO with thicknesses of 10–15 nm (Figure S7), in contrast to the significantly thicker BZYb20-5BaNiO_2_.

The statistical significance of the obtained Yb and Ni occupancies in BZYb20-5NiO is evaluated relative to a random distribution of the B-site constituents, described by a binomial distribution due to the discrete occupancies in columns with only 10–15 atoms (Figure S8). The obtained Yb and Ni occupancies lie beyond the expected values for the random distribution by a significant margin, i.e., consistent with point defect clustering.

### Nickel Point Defect Clusters Limit Proton Uptake

According to density functional theory (DFT) simulations, oxygen vacancies are trapped within the Yb–Ni clusters with a binding energy of −0.92 eV, calculated as the energy difference between placing the vacancy in the Yb–Ni cluster and at a distant lattice site. The formation of Yb–Ni clusters with trapped oxygen vacancies can be described according to

$${\mathrm{Yb}}_{\mathrm{Zr}}^{\prime }+{\mathrm{Ni}}_{\mathrm{Zr}}^{\prime }+v_{O}^{••}⇌{\left({\mathrm{Yb}}_{\mathrm{Zr}}^{\prime }v_{O}^{••}{\mathrm{Ni}}_{\mathrm{Zr}}^{\prime }\right)}^{\times }$$ 2

Assuming that all Ni reside in Yb–Ni clusters and considering the mass balance of Yb, the effective acceptor concentration of BZYb20 is determined by the amount of nonclustered Yb:

$${\left[{\mathrm{Yb}}_{\mathrm{Zr}}^{\prime }\right]}_{\mathrm{eff}}={\left[{\mathrm{Yb}}_{\mathrm{Zr}}^{\prime }\right]}_{\mathrm{tot}}-\left[{\left({\mathrm{Yb}}_{\mathrm{Zr}}^{\prime }v_{O}^{••}{\mathrm{Ni}}_{\mathrm{Zr}}^{\prime }\right)}^{\times }\right]={\left[{\mathrm{Yb}}_{\mathrm{Zr}}^{\prime }\right]}_{\mathrm{tot}}-{\left[{\mathrm{Ni}}_{\mathrm{Zr}}^{\prime }\right]}_{\mathrm{tot}}=\left[{\mathrm{OH}}_{O}^{•}\right]$$ 3

As shown in Figure 4a, the measured effective acceptor concentrations (hydration saturation limit) as a function of Ni content from BaNiO_2_ addition follows a linear relationship in agreement with the prediction according to eq 3. In other words, while each Ni acceptor leads to the formation of a half oxygen vacancy, each Ni traps one oxygen vacancy, thereby reducing the effective Yb acceptor concentration by one mole per mole of Ni.

**Figure 4:**
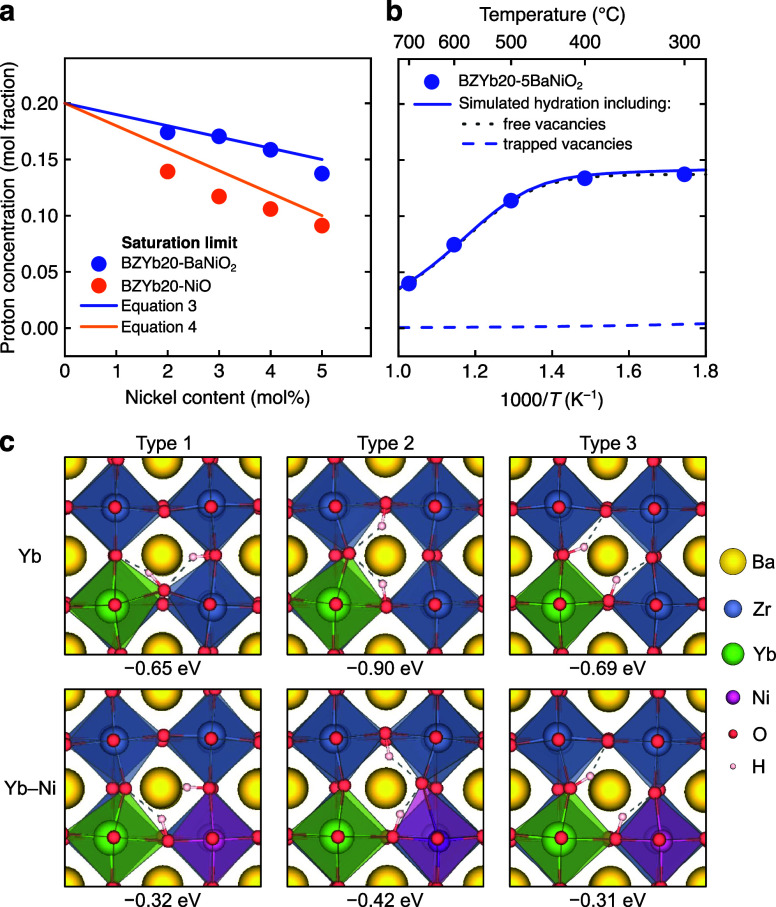
Hydration characteristics and saturation limits. (a) Proton saturation limit as a function of Ni content for BZYb20 with 2–5 mol % BaNiO_2_ or NiO. The solid lines correspond to the predictions according to eqs 3 and 4 with slopes of −1 and −2, respectively. (b) Hydration behavior of BZYb20-5BaNiO_2_ (*p*H_2_O = 0.02 atm) with contributions from the hydration of free oxygen vacancies (hydration entropy and enthalpy from Figure 1c) and trapped oxygen vacancies using the hydration enthalpy from DFT (−0.42 eV). (c) Hydration enthalpies of free oxygen vacancies, i.e., within (Zr_Zr_^×^v_O_^••^Yb_Zr_^′^), and oxygen vacancies trapped as (Yb_Zr_^′^v_O_^••^Ni_Zr_^′^) for three different configurations.

The hydration enthalpies of oxygen vacancies associated with Yb and Yb–Ni clusters are evaluated by DFT calculations, and the relaxed structures are summarized in Figure 4c. The mismatch in ionic radius between the primary Yb^3+^ and the small Ni^3+^ acceptors leads to pronounced local lattice distortions and a significantly less exothermic hydration enthalpy of oxygen vacancies in Yb–Ni clusters (−41 kJ/mol) compared with those associated with Yb (−86 kJ/mol). The simulated hydration behavior based on the obtained hydration enthalpies shows that the trapped oxygen vacancies manifest negligible hydration above 300 °C (Figure 4b), whereas kinetics limit further hydration at lower temperatures.^26^ Thus, the DFT simulations confirm that the trapped oxygen vacancies resist hydration and thereby support that the effective acceptor concentration can be described according to eq 3.

### Antiphase Boundaries Limit Proton Uptake

In the BZYb20-NiO system, antiphase boundaries (APBs) that disrupt the regular A- and B-site cation order and oxygen coordination environment are identified by nanoscale imaging (Figure 5a). The APBs form in the {111} planes and are associated with a lattice displacement of half a unit cell along [001] such that the A-site and B-site cations align across the boundary. The absence of Ba at the interface is confirmed by electron energy loss spectroscopy (EELS) (Figure 5b). These APBs, or stacking faults, thereby accommodate the excess B-site cations due to NiO addition. Notably, the APBs are significantly enriched in Yb, as is evident from the imaging contrast and EELS analysis (Figure 5b). The structure of the APB illustrated in Figure 5c, consistent with the HAADF and EELS images, is identified through DFT simulations as the most energetically favorable among APBs in the (001), (011), and (111) planes (Figure S9). The (001) and (111) APB structures are stabilized by the observed *a*/2 [001] lattice displacement when symmetry breaking is introduced through the rearrangement of Yb atoms.

**Figure 5:**
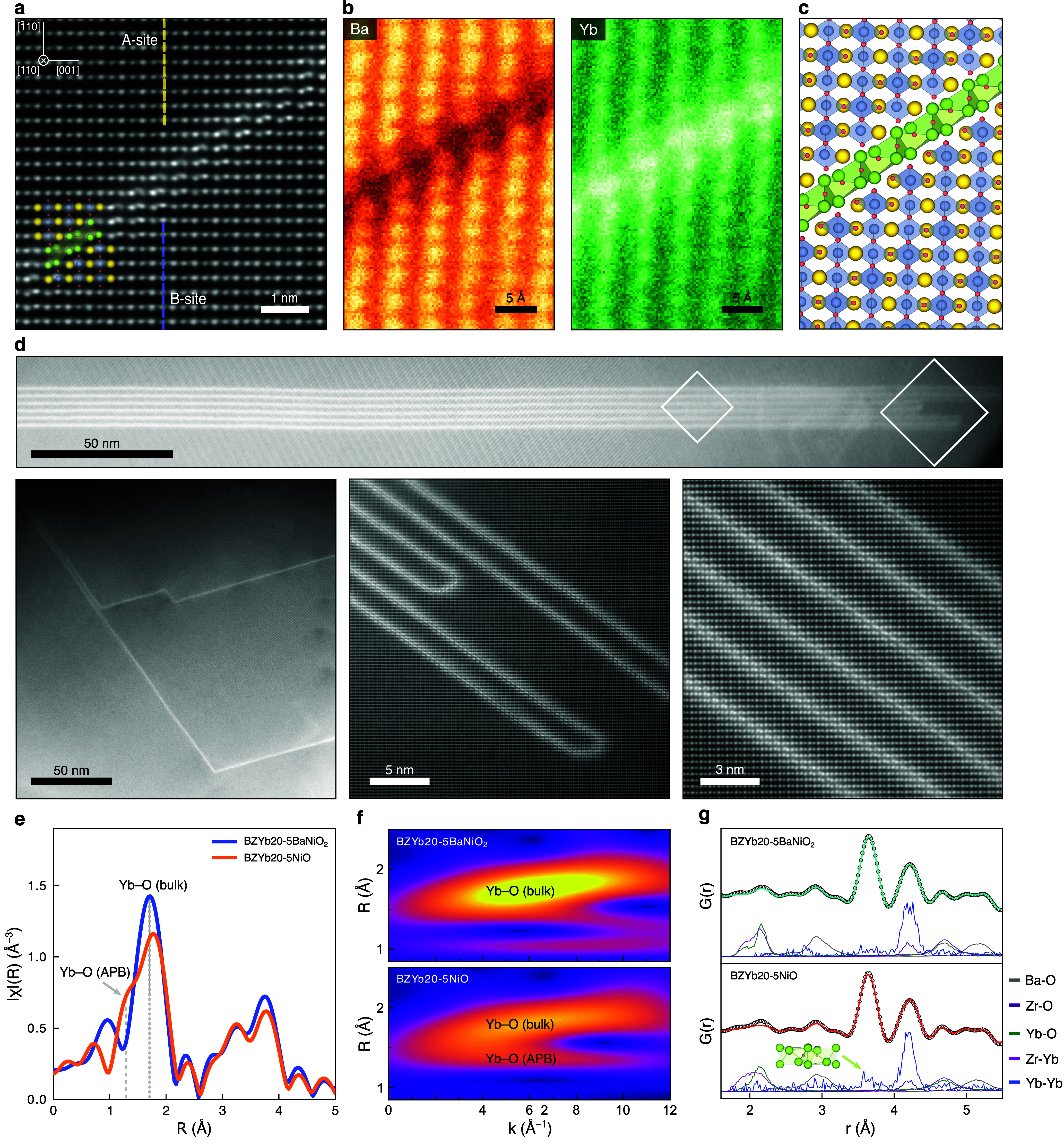
Nickel-induced formation of APBs in BZYb20-5NiO. (a) Atomic resolution STEM-HAADF images of an APB at the ⟨110⟩ zone axis. (b) EELS elemental maps of Ba and Yb at the APB. (c) Atomic structure model of the APB in the (111) plane with lattice translation vector a/2 [001]. (d) Overview STEM-HAADF images of APBs. (e) Fourier transform EXAFS spectra in R space. (f) Wavelet transform analysis of EXAFS spectra. (g) RMC fitting results and partial PDFs of selected atom pairs.

The APBs extend over the micrometer scale at all the investigated locations (Figure 5d), further supported by the appearance of a prominent shoulder in the Yb–O peak at approximately 1.8 Å in extended X-ray absorption fine structure (EXAFS) analysis (Figure 5e, with XANES and k space spectra in Figure S3). Wavelet transform analysis of the EXAFS data provides more detailed scattering contributions, highlighting the increased scattering intensity at approximately 1.8 Å associated with the shorter Yb–O bonds at the APB (Figure 5f). Although PDF analysis shows no significant difference between the two samples in either the local or average structure (Figure S10), Reverse Monte Carlo (RMC) simulations provide partial PDFs that reveal additional Yb–Yb pairs at approximately 3.6 Å in BZYb20-5NiO, corresponding to the first coordination shell associated with the APBs (Figure 5g and Figures S11–S12).

The formation of the APBs corresponds to the depletion of Yb acceptors from the bulk, further exacerbating the reduction in effective acceptor concentration beyond the Yb–Ni clustering. While it is challenging to quantify the extent of Yb depletion due to the APBs, it may be estimated based on stoichiometric considerations of the B-site excess in the BZYb20-NiO system. Here, it is assumed that the bulk cation nonstoichiometry is annihilated by the formation of APBs and that Yb is the main constituent of the APBs in line with the strong enrichment of Yb in the EELS analysis (Figure 5b). Accordingly, an amount of Yb equal to the NiO addition is depleted from the bulk, while Ni predominantly remains within the bulk, where it leads to point defect clustering and hydration suppression as described in eq 3. Thus, NiO addition exhibits dual effects on the effective acceptor concentration due to excess B-site cations leading to the formation of APBs as well as point defect clustering due to Ni substitution. Both contributions reduce the hydration saturation limit by a molar amount equal to the NiO addition, and the effective acceptor concentration in the BZYb20-NiO system can thereby be estimated according to

$${\left[{\mathrm{Yb}}_{\mathrm{Zr}}^{\prime }\right]}_{\mathrm{eff}}={\left[{\mathrm{Yb}}_{\mathrm{Zr}}^{\prime }\right]}_{\mathrm{tot}}-2{\left[{\mathrm{Ni}}_{\mathrm{Zr}}^{\prime }\right]}_{\mathrm{tot}}=\left[{\mathrm{OH}}_{O}^{•}\right]$$ 4

The estimated reduction in effective acceptor concentration of about two mol per mole NiO is in reasonable agreement with the measured hydration saturation limit for the BZYb20-NiO samples (Figure 4a). The formations of analogous APBs as those observed here have been reported in a minor phase of Ba_0.74_Zr_0.55_Y_0.45_O_3−δ_ within a Ba-deficient material with nominal composition Ba_0.9_Zr_0.8_Y_0.2_O_3−δ_, which also showed reduced hydration capability.^22^

## Discussion

Clustering of point defects is governed by stabilizing electrostatic interactions as well as relaxation of strain and local structural distortions. Trapping of oxygen vacancies and protons, both effectively positively charged, at acceptors is common for BaZrO_3_-based electrolytes.^27–29^ The resulting impact on proton uptake tends to be minor; e.g., the hydration enthalpy obtained here of oxygen vacancies within Yb–Yb clusters (−77 kJ/mol) is comparable to that of free oxygen vacancies (−86 kJ/mol) (Figure S13). In contrast, the Yb–Ni clusters predominantly exhibit oxygen vacancy trapping while protons are energetically unfavorable; i.e., the hydration enthalpy of trapped oxygen vacancies (−41 kJ/mol) is significantly suppressed.

To further elucidate the scope of Ni-induced point defect clustering in BaZrO_3_, the trapping and hydration characteristics of oxygen vacancies in clusters with Ni and other trivalent acceptors (Ga, Sc, In, and Y) were evaluated by DFT (Figure S14). Analogous to the case with Yb, these acceptors also form stabilized clusters with Ni, which all exhibit suppressed hydration enthalpies. These findings indicate Ni-induced hydration suppression to be universal in acceptor substituted BaZrO_3_ and presumably other systems.

Beyond suppressing the proton concentration, Yb–Ni clusters may impact proton mobility by disrupting the acceptor percolation pathways that facilitate proton migration.^30–32^ The presence of APBs may further impact bulk proton mobility, acting as resistive interfaces within grains. The combined effects of Ni on proton concentration and mobility may thereby account for the more than 1 order of magnitude reduction in bulk conductivity observed in Y/Yb-doped BaZrO_3_ sintered with NiO.^9^

The detrimental impact of Ni on the bulk protonic conductivity in BaZr_0.8–*x*_Ce_*x*_Y_0.2_O_3−δ_ (0 ≤ *x* ≤ 0.8) sintered with 2 wt % NiO systematically diminishes with increasing Ce content.^12^ This indicates that Ce substitution mitigates Ni-induced point defect clustering and APB formation. Indeed, introduction of 1 wt % NiO (∼4 mol %) into BaZr_0.4_Ce_0.4_Y_0.1_Yb_0.1_O_3−δ_ or BaZr_0.4_Ce_0.4_Y_0.2_O_3−δ_ ^20^ yields a higher hydration saturation limit of approximately 15 mol % compared to 11 mol % obtained here for BZYb20-4NiO (Figure 4a), while the origin of these differences remains the subject of future investigations.

In summary, this study reveals that Ni-induced point defect clustering suppresses the hydration of oxygen vacancies in BaZrO_3_-based electrolytes. Introduction of excess B-site cations through NiO additives further suppresses hydration by forming antiphase boundaries that accumulate acceptors. These findings highlight the critical role of Ni diffusion from the electrode into the electrolyte during cosintering and pave the way for defect engineering approaches to realize the inherent potential of BaZrO_3_-based electrolytes.

## Methods

### Materials

BaZr_0.8_Yb_0.2_O_2.9_ with *x* mol % NiO and BaNiO_2_ (*x* = 2, 3, 4, 5) were prepared using the solid-state reactive sintering method from the following raw materials: BaCO_3_ (99% purity, Sigma-Aldrich, Germany), Yb_2_O_3_ (99.9% purity, Alfa Aesar, Germany), NiO (Ni 78.5%, Alfa Aesar, Germany), and ZrO_2_ (99.9% purity, Sigma-Aldrich, Germany). Stoichiometric powder mixtures were roll milled in 2-isopropanol. After evaporation of 2-isopropanol, the dried powders were uniaxially pressed into disks under a pressure of 150 MPa and sintered at 1500 °C for 5 h in ambient atmosphere. Powder samples were obtained by grinding the sintered pellets, and their phase purities were confirmed by X-ray diffraction using a D8 Advance Diffractometer (Bruker, USA) with Cu Kα radiation (Figure S1).

### Thermogravimetric Analysis

Thermogravimetric analyses were performed using a 449 F1 Jupiter thermal analyzer (Netzsch GmbH, Germany). The powder samples were first degassed by heating to 900 °C at a rate of 5 °C min^–1^ under flowing bottle-dry N_2_ (99.999%) until the sample mass stabilized. Subsequently, the isothermal water uptake was measured under humidified N_2_ (*p*H_2_O = 0.02 bar) during stepwise cooling from 700 to 100 °C (Figure S2).

### X-ray Total Scattering and Absorption Spectroscopy

Synchrotron total scattering with X-ray diffraction and X-ray absorption spectroscopy (XAS) experiments were performed at the Swiss-Norwegian beamline BM31 at the European Synchrotron Research Facilities (ESRF).^34^ Quartz capillaries with a diameter of 0.3 mm filled with powder were mounted with back-and-forth movement during measurements at room temperature for total scattering measurements. An X-ray with a wavelength λ = 0.25995 Å was applied, and total scattering patterns were measured using a PILATUS3 X CdTe 2M detector for diffraction and a pair distribution function (PDF). Analysis of the diffraction and PDF data was carried out with TOPAS v6 Academic^35^ and “small-box” modeling, with PDFgui^36^ and “big-box” modeling RMCProfile software packages.^37^ XAS measurements were performed at the Ni K edge (8333 eV) and Yb L3 edge (8944 eV) in transmission mode. Energy calibration was performed using Ni foil placed downstream of the sample, and the obtained data were processed and analyzed using the Demeter suite of programs.^38^

### Scanning Transmission Electron Microscopy

Scanning transmission electron microscopy (STEM) was performed on an FEI Titan G2 60-300 instrument with a DCOR Cs probe corrector operated at 200 kV with a probe current of 0.1 nA controlled with a Wien-filter monochromator. Powder samples were mortared in high-purity isopropanol and drop-casted on a copper grid with a lacey carbon film. The imaging optics were optimized for high-angle annular dark-field (HAADF) imaging with a convergence semiangle of 25.1 mrad and an inner collection angle of 66.6 mrad, calibrated from a standard Agar Scientific S106 Au diffraction grating (Figure S7). The images were postprocessed using the drift-corrected frame integration algorithm in the Velox software, and the convolution neural network was developed by Lobato et al.^39^ The abTEM package^25^ was used to simulate the STEM-HAADF images from ⟨120⟩ orientated supercells of 12 nm thickness, constructed using the Atomic Simulation Environment package,^40^ containing 2, 4, and 6 Yb–Ni clusters and two Yb atoms randomly distributed on the other B-site columns (occupancy 0.17). The probe and detector collection angles were matched to the calibrated values in the experiment. The atomic potentials were modeled using Kirkland parametrization^41^ with 2 pm sampling, frozen phonons averaging 50 configuration with 0.1 Å standard deviation for the displacements, and a slice thickness of 0.5 Å. The simulated images were interpolated linearly with three additional points between existing pixels and then smoothed with a Gaussian filtered standard deviation of 6 pixels prior to image analysis. Analysis of the atomic columns was handled with Atomap^42^ to refine positions, perform integration using the Voronoi method, and normalize the integrated intensities of the Yb- and Ni-enriched columns to the B-site reference. The overall uncertainty of the Yb and Ni occupancies was estimated based on random and systematic error in the experiment and data processing (Table S2).

Electron energy loss spectroscopy (EELS) was performed using a Gatan Quantum ER 965 spectrometer with a convergence angle and collection aperture of 25.5 and 49.7 mrad, respectively. Data handling was performed in the exSPy ecosystem of the Hyperspy package.^43^ The spectral images were denoised using principal component analysis^44^ prior to background removal using a second order polynomial function and integration of the M-edges of Ba and Yb to obtain elemental maps. The thickness map of the BZYb20-5NiO sample was calculated using the log-ratio method on the low-loss EELS region^45^ (Figure S7).

### Statistical Analysis of Random Yb and Ni Distributions

Statistical analysis was performed by calculating the probability of occupancy from a binominal distribution:

$$P\left(k\right)=\left(\begin{array}{c} n\\ k \end{array}\right)\;p^{k}{\left(1-p\right)}^{n-k}$$ 5

Here, *P*(*k*) is the probability of observing *k* specific atoms in a column of *n* atoms with probability *p* of occupancy taken from the nominal molar fraction of the B-site. The binominal distribution was chosen due to the small number of atoms in the columns, i.e., below 30 according to the central limit theorem, resulting in discrete occupancies. The joint probability of Yb and Ni occupancy in separate groups was calculated by multiplying their individual probabilities since filling of lattice sites are independent events in a random distribution. The sensitivity of the binomial distribution to variations in thickness across the selected regions was evaluated by changing *n* between 10 and 15, corresponding to thicknesses of approximately 10–15 nm (Figure S8).

### DFT Simulations

DFT calculations were performed using the Vienna Ab initio Simulation Package (VASP) with the PBE generalized gradient approximation.^46,47^ BaZrO_3_ was taken to represent the host structure, and all calculations involving different dopants and defects were simulated in 4 × 4 × 4 supercells of the cubic unit cell (135 atoms). The standard PBE PAW potentials supplied by VASP were used, including Ba_sv (5s^2^5p^6^6s^2^), Zr_sv (4s^2^4p^6^4d^2^5s^2^), Ni (3d^8^4s^2^), O (2s^2^2p^2^), and H (1s). The Yb_3 (5p^6^6s^2^5d^1^) pseudopotential for Yb was used here as Yb_2 (5p^6^6s^2^) gives incorrect thermodynamics for some systems with Yb^3+.^ ^48^ The plane wave energy cutoff was set to 500 eV, and the Brillouin zone was sampled at the Γ-point. The ionic positions and electronic structure were optimized with convergence criteria of −0.02 eV Å^–1^ and 10^–6^ eV, respectively.

The hydration energies (*E*_hyd_) were calculated by subtracting the total energies of the water-free doped structure (*E*_doped_^tot^) and an isolated water molecule (*E*_water_^tot^) from the total energy of the corresponding hydrated doped structure (*E*_hydrated_^tot^):

$$E_{\mathrm{hyd}}=E_{\mathrm{hydrated}}^{\mathrm{tot}}-E_{\mathrm{doped}}^{\mathrm{tot}}-E_{\mathrm{water}}^{\mathrm{tot}}$$ 6

Here, the doped structure included the isolated point defect of oxygen vacancies created by substitution of an Yb associated with Ni reacted with water through the hydration reaction (eq 1).

## Supplementary Material



## Data Availability

Data and analysis procedures are available in the Zenodo repository at 10.5281/zenodo.17305651.

## Notes

Phase purity (XRD), hydration thermodynamics (TG), structural characterization (PDF and XAS), nanoscale imaging of Yb–Ni clusters (STEM), statistical analysis of Yb–Ni occupancies (STEM), estimation of error and uncertainty (STEM), antiphase boundary structures and energetics (DFT and PDF), and hydration energetics of dopant clusters (DFT) (PDF)
