# Supplementary material for: Nickel-Induced Lattice Defects Limit Proton Uptake in Barium Zirconate Electrolytes
Source: J Am Chem Soc. 2025 Dec 12;148(1):379–87. doi: 10.1021/jacs.5c13935 (PMC12814359; doi:10.1021/jacs.5c13935)
Supplement: Supplementary file 1 [file ja5c13935_si_001.pdf]

Supporting Information

Nickel-induced lattice defects limit proton uptake in barium zirconate electrolytes

Yabing Wen[1,†], Andreas Rosnes[2,†], Bo Jiang[1,*], Øystein Prytz[2], Truls Norby[1], Reidar Haugsrud[1,*], Jonathan M. Polfus[1,*]

[1] Department of Chemistry, Centre for Materials Science and Nanotechnology, University of Oslo, PO Box 1033 Blindern, NO-0315 Oslo, Norway

[2] Department of Physics, Centre for Materials Science and Nanotechnology, University of Oslo, PO Box 1048 Blindern, NO-0316 Oslo, Norway

† Equally contributing authors

* Corresponding authors email: bo.jiang@kjemi.uio.no; reidar.haugsrud@kjemi.uio.no; jonathan.polfus@kjemi.uio.no

Table of Contents

S1. Phase purity (XRD) 2
S2. Hydration thermodynamics (TG) 2
S3. Structural characterization (PDF and XAS) 4
S4. Nanoscale imaging of Yb-Ni clusters (STEM) 5
S5. Statistical analysis of Yb-Ni occupancies (STEM) 7
S6. Estimation of error and uncertainty (STEM) 8
S7. Anti-phase boundary structures and energetics (DFT and PDF) 9
S8. Hydration energetics of dopant clusters (DFT) 12

S1. Phase purity (XRD)

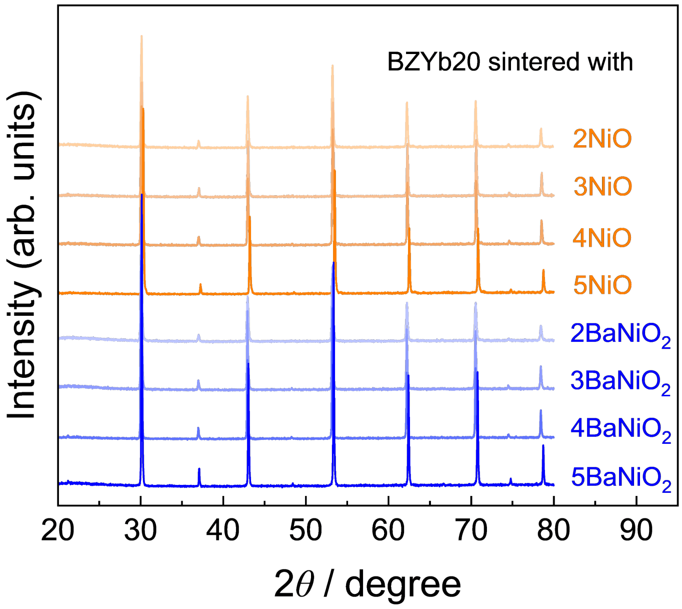

Figure S1. X-ray diffraction data of $BaZr_{0.8}Yb_{0.2}O_{2.9}$ sintered with x mol% NiO or $BaNiO_2$ (x = 2, 3, 4, 5).

S2. Hydration thermodynamics (TG)

The hydration profiles (Fig. 1c) are fitted according to the procedure by Kreuer [1]:

$$[\mathrm{OH_O^{\bullet}}]=\frac{-3+\sqrt{9+S\cdot(6-S)\dfrac{4-p\mathrm{H_2O}\cdot K}{p\mathrm{H_2O}\cdot K}}}{\dfrac{4-p\mathrm{H_2O}\cdot K}{p\mathrm{H_2O}\cdot K}} \qquad (1)$$

where $[\mathrm{OH_O^{\bullet}}]$ is the molar fraction of protonic defects, S represents the effective acceptor level (mole fraction), and K is the equilibrium constant of the hydration reaction, expressed in terms of the standard hydration enthalpy and entropy as

$$K=\exp\left(-\frac{\Delta H^{\circ}-T\Delta S^{\circ}}{RT}\right) \qquad (2)$$

The thermodynamic parameters and effective acceptor levels obtained from fitting the proton concentration data (Fig. 1c) are listed in the Table S1.

Table S1. Thermodynamic parameters of hydration (ΔH° and ΔS°) and effective acceptor levels S of $BaZr_{0.8}Yb_{0.2}O_{2.9}$ sintered with x mol% NiO or $BaNiO_2$ (x = 2, 3, 4, 5).

Sample	ΔH° (kJ/mol)	ΔS° (J/mol K)	S (mole fraction)
BZYb20-2NiO	−88.3±7.4	−95.3±8.5	0.138
BZYb20-3NiO	−98.3±6.5	−107.8±7.5	0.116
BZYb20-4NiO	−96.6±7.2	−106.9±8.4	0.106
BZYb20-5NiO	−99.1±6.6	−109.4±7.7	0.091
BZYb20-2BaNiO$_2$	−92.1±7.6	−102.6±8.8	0.174
BZYb20-3BaNiO$_2$	−97.5±7.2	−107.4±8.4	0.170
BZYb20-4BaNiO$_2$	−105.3±7.1	−114.4±8.2	0.159
BZYb20-5BaNiO$_2$	−108.5±5.3	−116.0±6.1	0.137

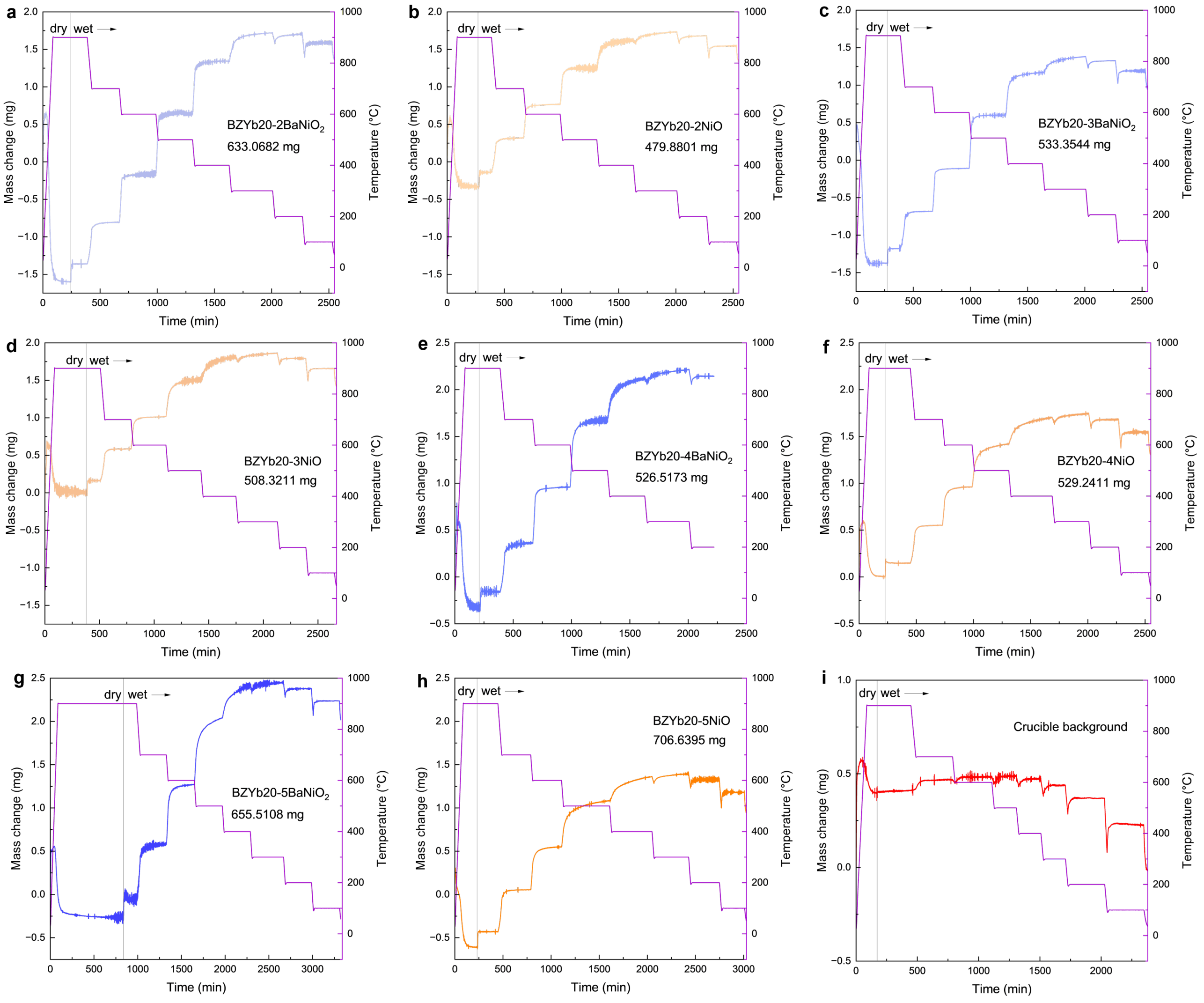

Figure S2. Thermogravimetric analysis of hydration of powder samples (mass given in the figures) and background measurement with empty crucible. The isothermal water uptake was measured under humidified N_2 (pH_2O = 0.02 bar) during stepwise cooling from 700 °C to 100 °C with a dwell of approx. 5 hours to ensure complete equilibration. Hydration saturation is achieved below approx. 300°C where the slight reduction in mass due to buoyancy is corrected by background measurement.

S3. Structural characterization (PDF and XAS)

Notably, the effects of NiO and $BaNiO_2$ appear to be similar, without significant differences in their impact on the local and average structure (Figure S3a). The XANES profiles confirm that both samples exhibit a similar Yb^{3+} oxidation state (Figure S3b). A comparison of the EXAFS spectra (Figure S3c) and the Fourier-transformed (FT) R-space data (Figure 5e) at the Yb L3-edge, reveals a comparable local structural environment for Yb in both samples.

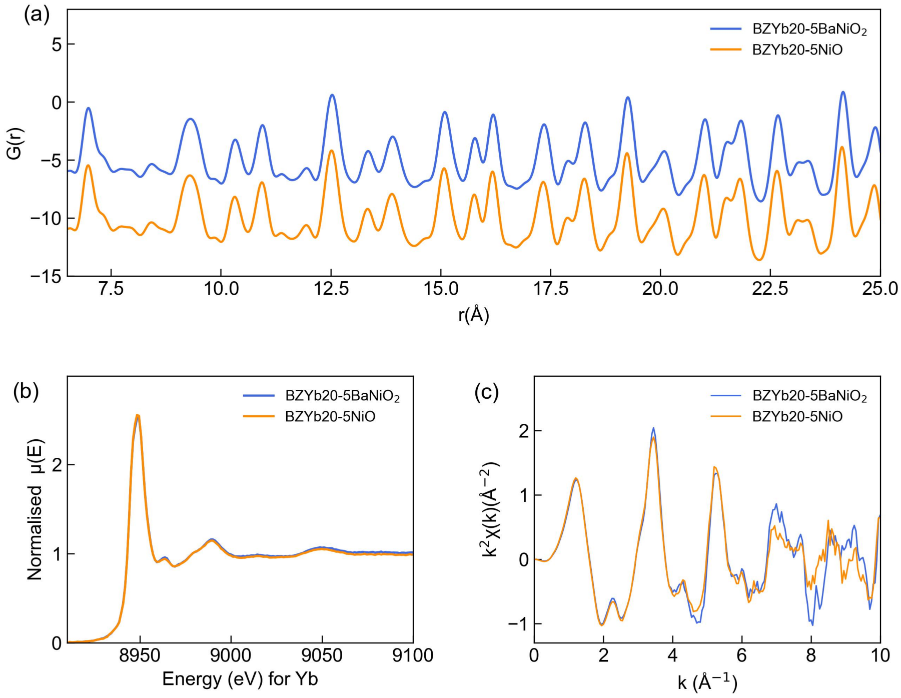

Figure S3. a, X-ray pair distribution function of BZYb20-5BaNiO$_2$ and BZYb20-5NiO at medium structure range. **b,** XANES spectra and **c,** EXAFS spectra in k space of Yb L3-edge.

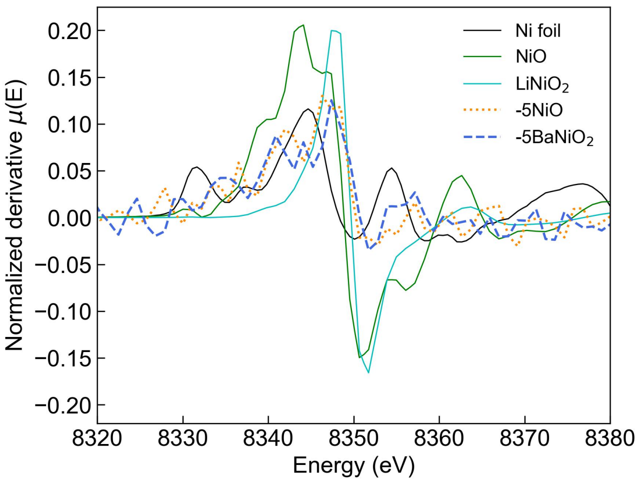

Figure S4. First derivative XANES spectra of BZYb20-5BaNiO$_2$, BZYb20-5NiO and references for Ni foil, NiO and LiNiO$_2$.

S4. Nanoscale imaging of Yb-Ni clusters (STEM)

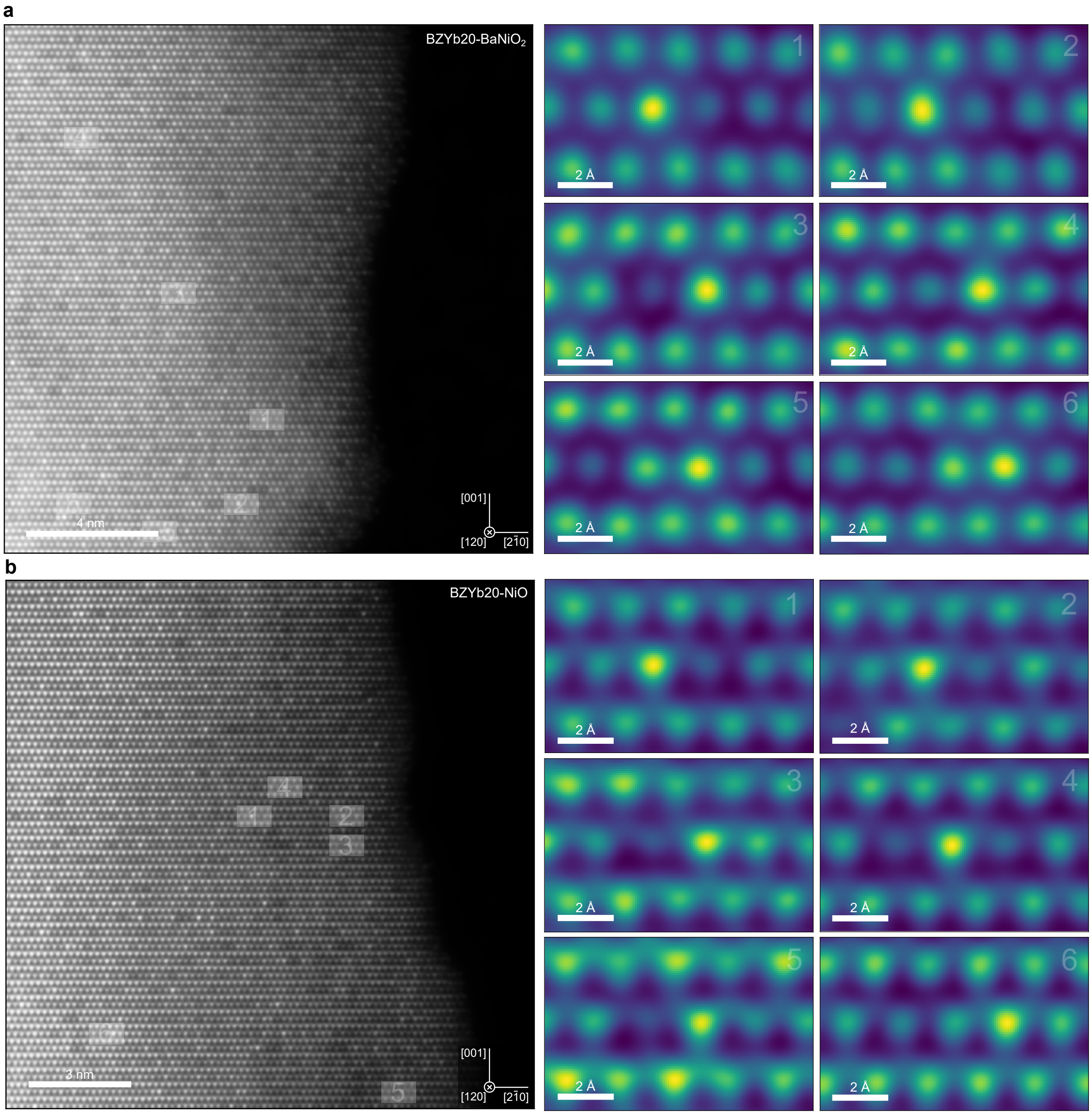

Figure S5. a, Atomically resolved <120> oriented STEM-HAADF images of BZYb20-5BaNiO$_2$ and **c,** BZYb20-5NiO show inhomogeneous intensity distribution on the B-site. **b, d,** In multiple regions, dark atomic columns are found adjacent to bright columns, indicative of neighbouring columns enriched in Ni and Yb, respectively.

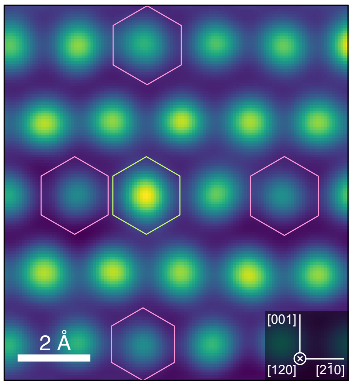

Figure S6. Atomically resolved <120> oriented STEM-HAADF image of BZYb20-5$BaNiO_2$ with Yb clustered with Ni at nearest and second nearest neighbour columns along [2$\bar{1}$0] and in each direction along [001].

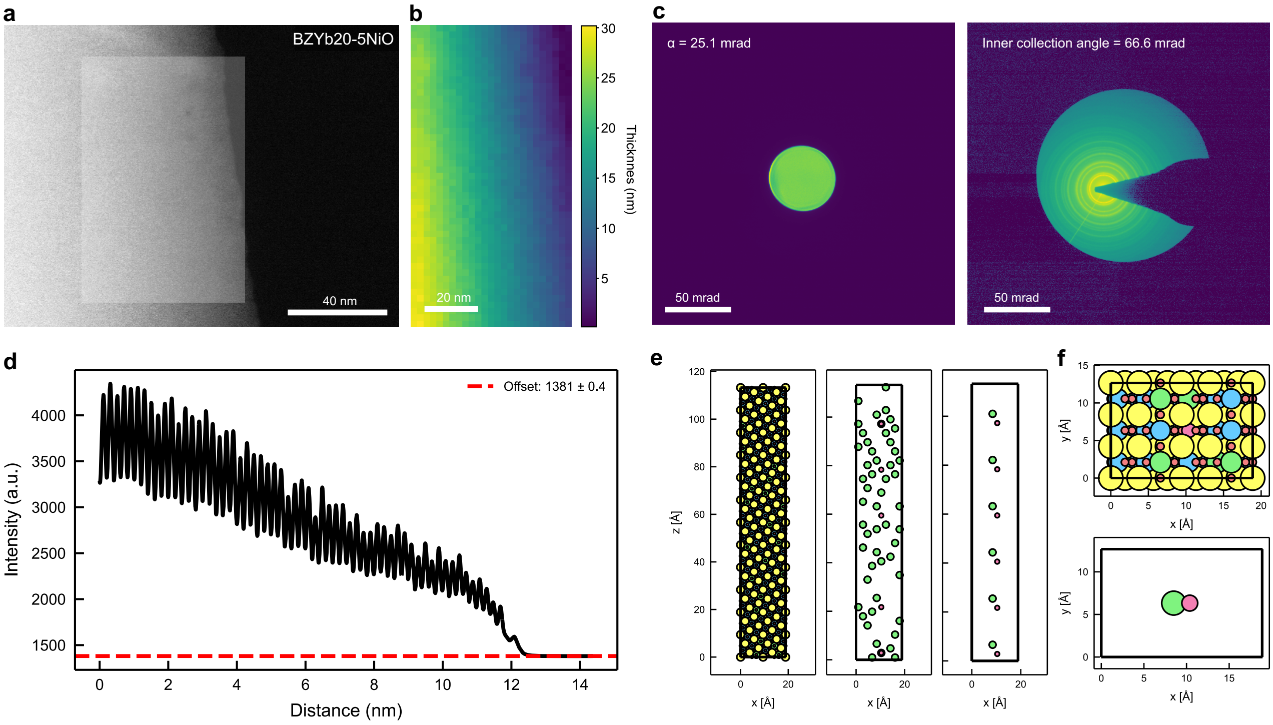

Figure S7. a, Overview STEM HAADF image of the studied wedge of the BZYb20-5NiO sample. **b,** Thickness map obtained from low-loss EELS signal using the log-ratio method of the shaded region in **a**. **c,** STEM image in the back-focal plane showing the bright field disk, inner radius of the HAADF detector, and the Debye rings from a gold refence sample. **d,** Line profile across the Ba sites used to identify the offset value in vacuum. **e,** Sideview of supercell with all atoms (left), six Yb-Ni clusters and randomly distributed Yb (middle), and only the six Yb-Ni clusters (right). **f,** Top view of the supercell with all atoms (top) and only Yb-Ni clusters (bottom).

S5. Statistical analysis of Yb-Ni occupancies (STEM)

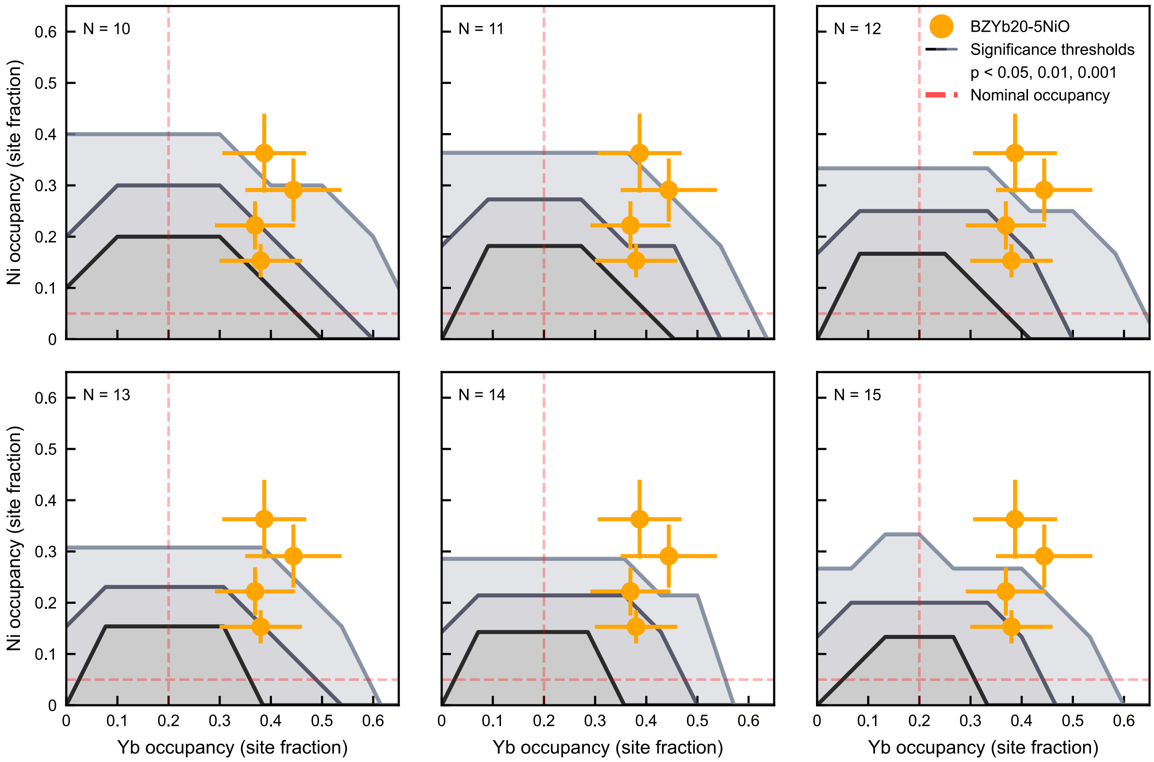

Figure S8. Occupancies of Yb and Ni from the relative intensities of the Voronoi integration of selected regions in BZYb20-5NiO. The overlayed map shows threshold lines for the statistical significance (p-values) of Yb and Ni occupancy at neighbouring columns from binomial distributions with 10–15 atoms in the columns.

S6. Estimation of error and uncertainty (STEM)

The uncertainty of the Yb and Ni occupancy is evaluated from a combination of random and systematic errors from the experiment and analysis approach, summarized as follow:

- Random error from the counting statistics of the detector was calculated based on the square root of the number of counts for the Yb and Ni rich columns, as well as the B-site used as normalization factor.
- Systematic error due to non-linearity from the asymmetry of the Fischione 3000 ADF detector is included as the worst-case scenario of 15% of the intensity since the detector flux was not calibrated [2]. The error combines with that of the counting statistics in quadratures.
- The propagation of error to obtain the relative intensity by division of the intensity of the Yb and Ni rich columns with the normalization factor are calculated according to equation (3).

$$\frac{\delta I_{\mathrm{Rel.}}}{|I_{\mathrm{Rel.}}|} = \sqrt{\left(\frac{\delta I_{\mathrm{Yb,Ni}}}{I_{\mathrm{Yb,Ni}}}\right)^2 + \left(\frac{\delta I_{\mathrm{B-site}}}{I_{\mathrm{B-site}}}\right)^2} \tag{3}$$

- Error due to instability, drift and instrumental noise is limited by utilizing a fast scan speed, and drift-correction and denoising in the post-processing, and thus neglected due to the relatively minor contribution compared to other sources of error.
- Errors due to improper amplifier and brightness settings of the detector were minimized by observing the waveform during acquisition and adjusting accordingly [2].
- Intensity variation due to sample tilt was minimized by only employing intensity measurements on local pairs of atomic columns where the region contains negligible variation in tilt [2].
- The linear fit to the relative intensities of the simulated images is used to convert the integrated intensities of the measurements to occupancy. The uncertainty in predicting an unknown occupancy using the linear extrapolation is calculated from the standard error [3] of the prediction as in equation (4).

$$SE_{\mathrm{pred}}(x) = s\sqrt{1 + \frac{1}{n} + \frac{(x-\bar{x})(x-\bar{x})^2}{\sum_{i=1}^{n}(x_i-\bar{x}_i)^2}} \tag{4}$$

  Here, s is the standard error of the regression, n is the number of data points, and x and $\bar{x}$ are the measured and predicted values, respectively. The relative intensity is converted to occupancy by dividing by the scaling factor, where the error propagates similarly as in equation (3).

The obtained occupancies with the total estimated uncertainties are summarized in Table S2 for the analysis of the selected regions with the contribution of the random and systematic error in percentage of the total uncertainty.

Table S2. Occupancy of Yb and Ni with total absolute uncertainty in the selected regions in BZYb20-5NiO and the contributing random and systematic errors used for semi-quantitative analysis. *The normalization factor is a combined random error and systematic error from the counting statistics and detector calibration.

Region	Element	Occupancy	Random error (%)	Systematic error (%)		
			Counts	Normalization*	Detector	Scaling
1	Yb	0.39±0.08	<0.001%	49.98%	49.98%	0.04%
	Ni	0.36±0.08	<0.001%	49.93%	49.93%	0.14%
2	Yb	0.44±0.09	<0.001%	49.98%	49.98%	0.04%
	Ni	0.29±0.06	<0.001%	49.93%	49.93%	0.14%
3	Yb	0.37±0.08	<0.001%	49.98%	49.98%	0.14%
	Ni	0.22±0.05	<0.001%	49.93%	49.43%	0.04%
4	Yb	0.38±0.08	<0.001%	49.98%	49.98%	0.14%
	Ni	0.15±0.03	<0.001%	49.93%	49.93%	0.04%

S7. Anti-phase boundary structures and energetics (DFT and PDF)

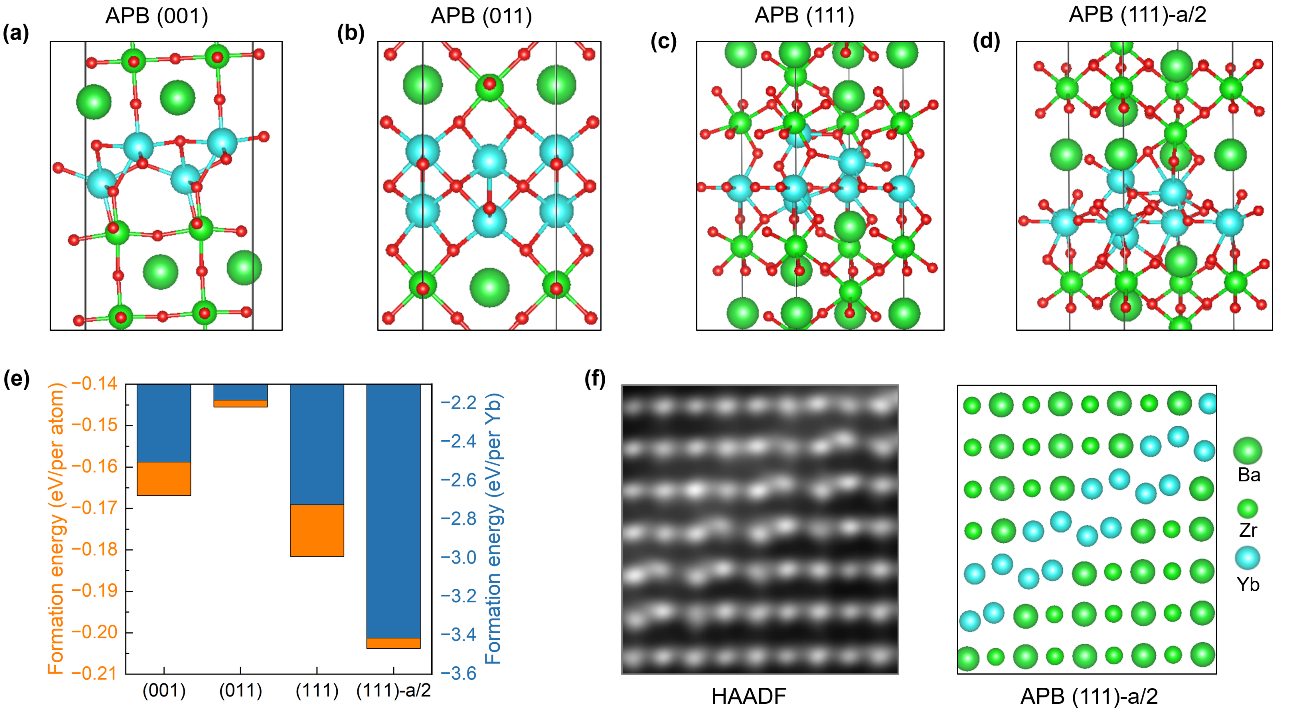

Figure S9. DFT simulations of APB configurations. a, APB (001), **b,** APB (011), **c,** APB (111), and **d,** APB (111) with ½ unit cell displacement between adjacent regions in APB. **e,** Calculated relative energy of different APBs. **f,** Comparison of the HAADF-STEM image and APB (111) with ½ unit cell displacement. Positional symmetry breaking was applied to all models by shifting a few atoms from their ideal positions with the ISYM=0 setting.

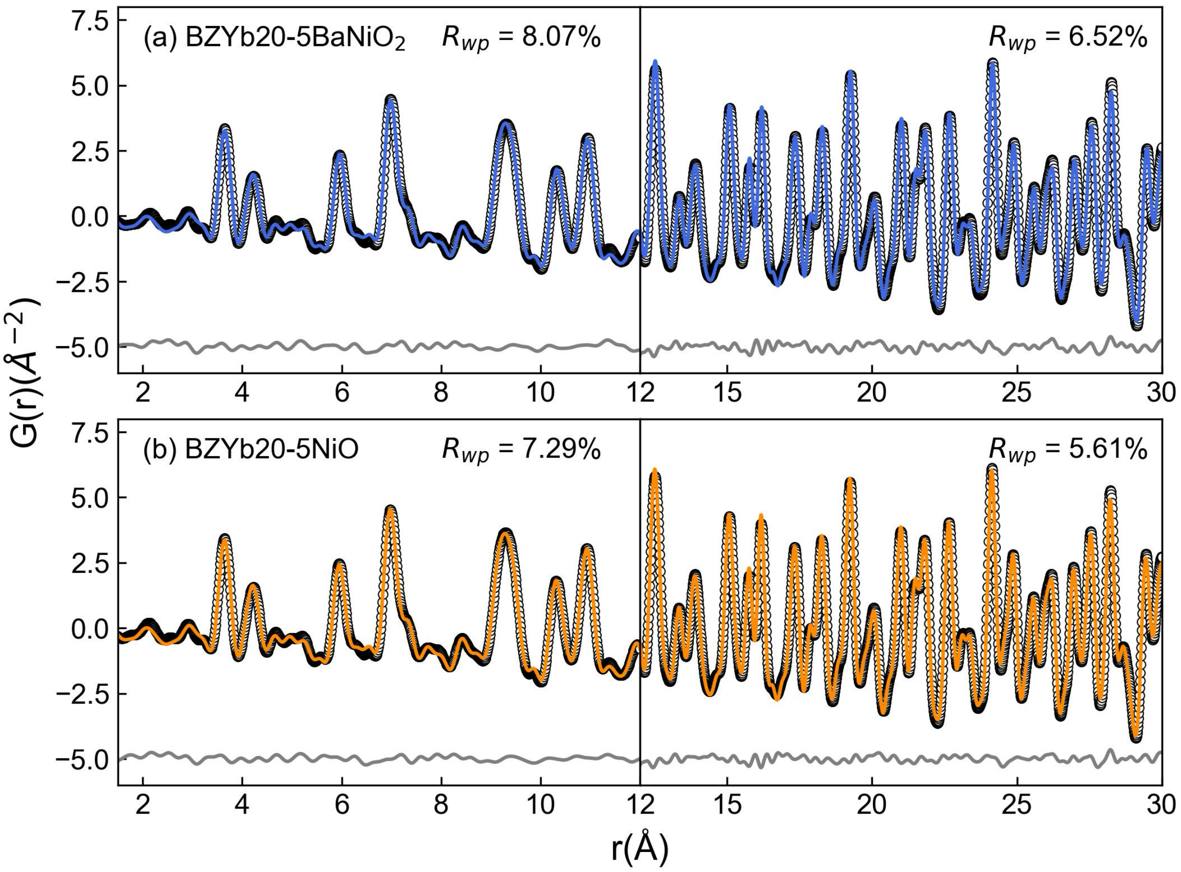

Figure S10. Results of 'small-box' PDFgui refinements of X-ray PDFs over local (1.5–12 Å) and medium range (12–30 Å) to (a) BZYb20-5BaNiO$_2$ and (b) BZYb20-5NiO using the cubic Pm$\bar{3}$m structure.

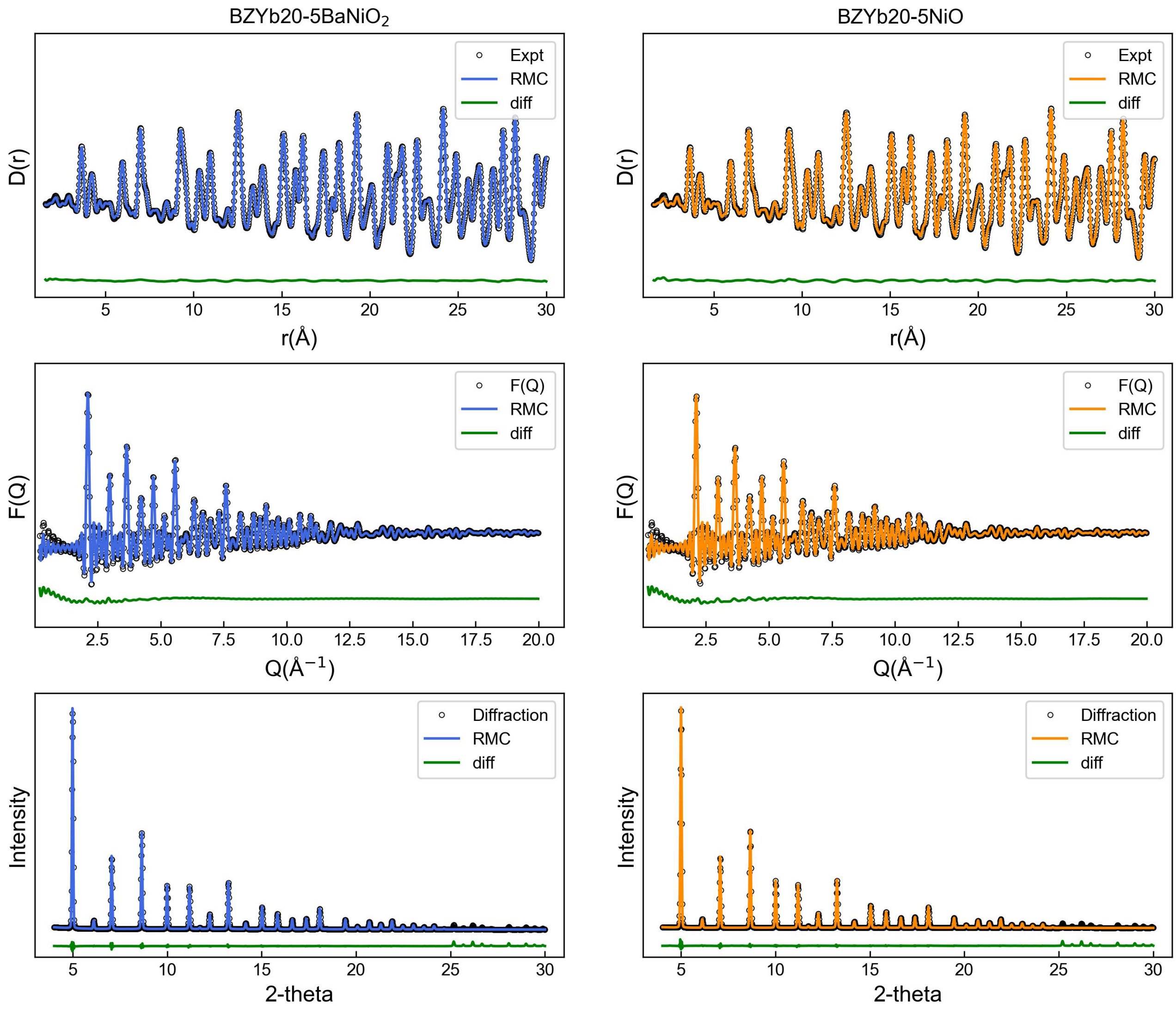

Figure S11. Total scattering fits to D(r), F(q) and Bragg data with atom swapping from RMC simulations for BZYb20-5BaNiO_2 (left), and BZYb20-5NiO (right).

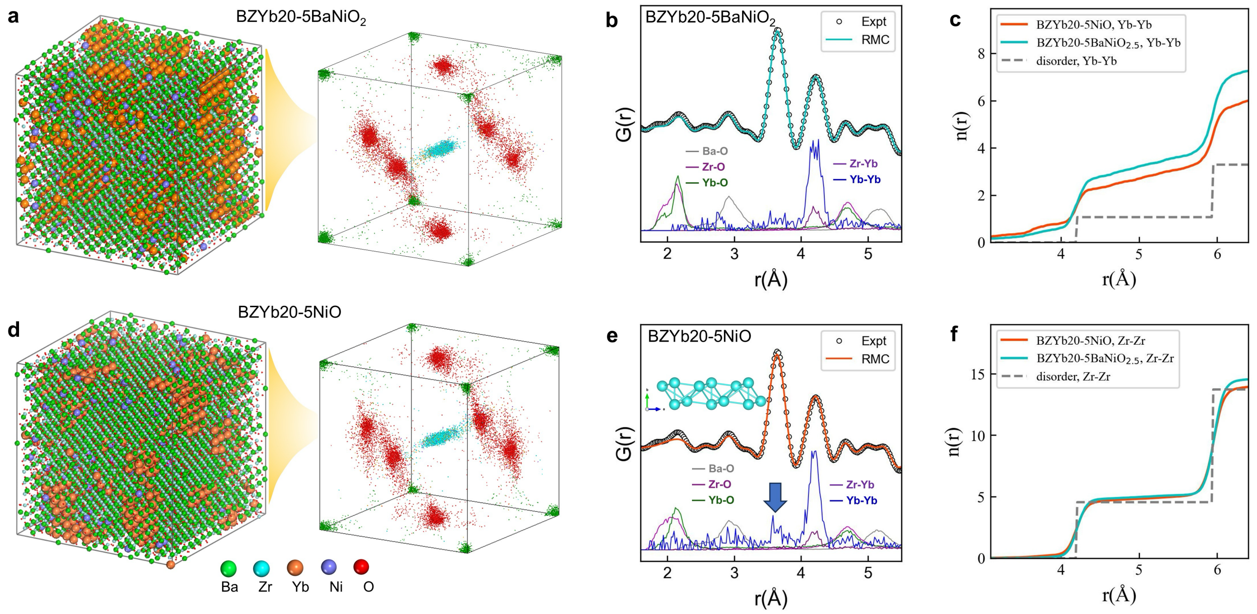

Figure S12. a, d A 15 × 15 × 15 supercell RMC model after $1*10^7$ moves and the corresponding folded cubic unit cell on the right showing the atomic distribution cloud. The Yb shows an ellipsoid-shaped, uniform distribution of atoms, confirming the local chemical ordering of Yb-Yb pairs. **b, e** RMC fitting results and selected partial PDFs showing the atom pairs below. **c, f** Nearest neighbour functions n(r) displaying the local chemical ordering (LCO) of Yb-Yb and Zr-Zr cation pairs resulting from RMC modelling for BZYb20-5BaNiO$_2$ and BZYb20-5NiO, the dashed grey lines show the theoretical value from the disordered structure.

S8. Hydration energetics of dopant clusters (DFT)

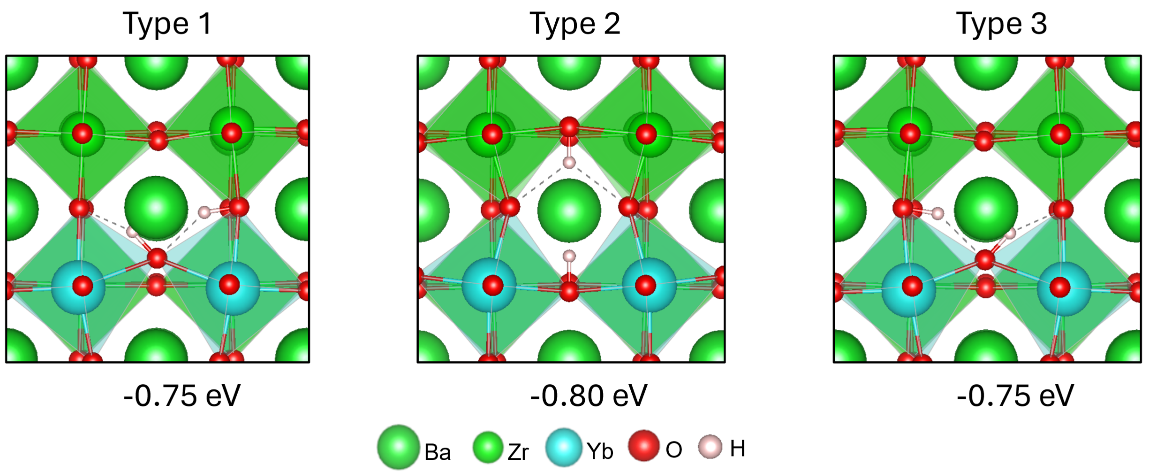

Figure S13. Hydration enthalpies of oxygen vacancies trapped within $Yb'_{Zr}v_O^{\bullet\bullet}Yb'_{Zr}$ for three different configurations.

Density functional theory (DFT) calculations were performed on different configurations of the $BaZrO_3$ supercell addressing potential associations between M^{3+} and Ni^{3+} (M = Ga, Sc, In, Yb, Y), where the $Ni'_{Zr} - v_O^{\bullet\bullet} - M'_{Zr}$ configurations were verified to be the most stable (Figure S14a-c). The relaxed hydrated structures of these clusters exhibit generally less negative hydration energy (enthalpy) compared to the that of the Ni-free BZYb system (Figure S14d-e).

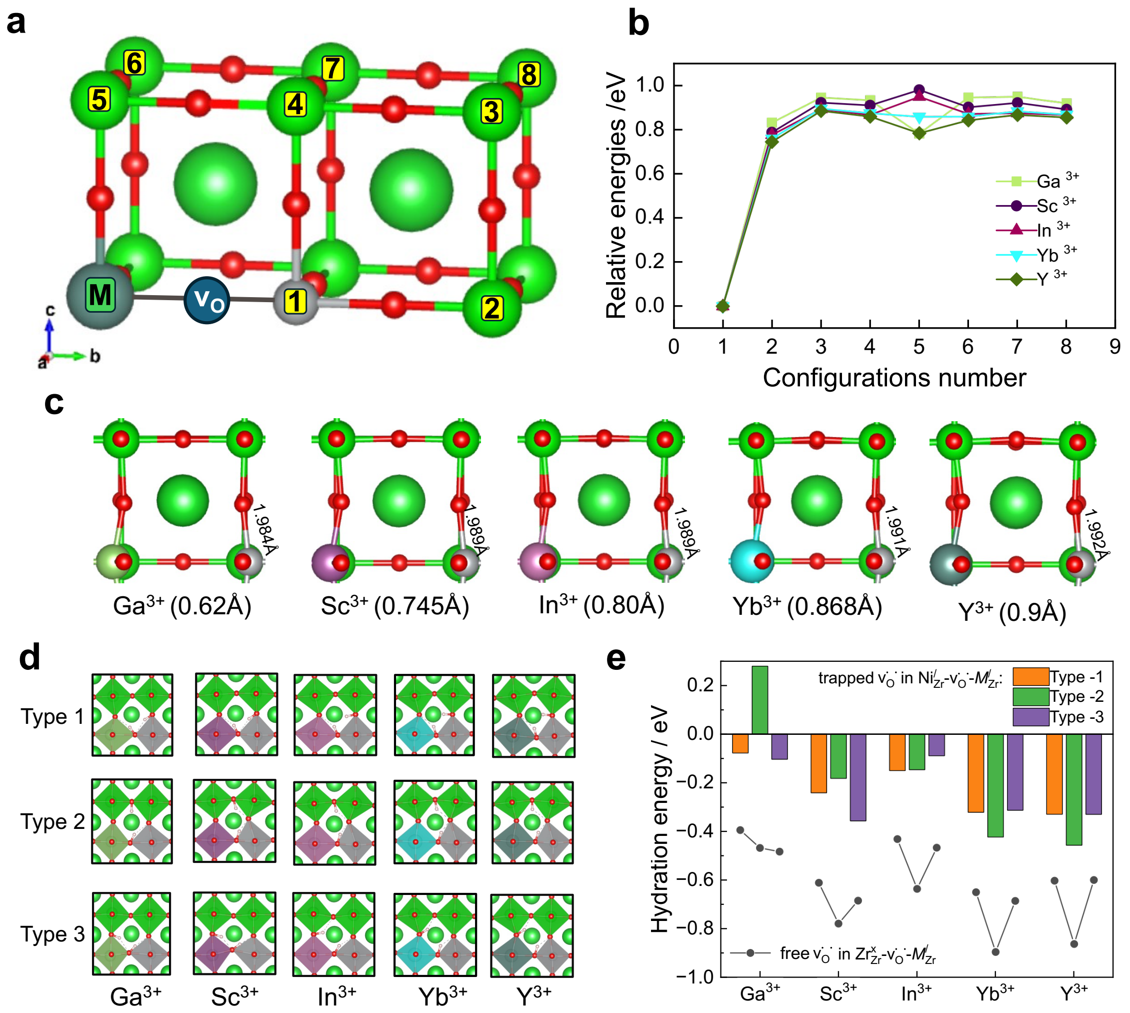

Figure S14. DFT simulations of Ni-induced point defect clustering with M cations (M = Ga, Sc, In, Yb and Y) in $BaZrO_3$. **a,** Representative nickel cation configurations near the oxygen vacancies in a $BaZrO_3$ supercell doped with M cations, illustrating as $M'_{Zr}v_O^{\bullet\bullet}$ defect. **b,** Relative total energies of different configurations number for M cations doped after DFT relaxation. **c,** Fragments of $M'_{Zr}v_O^{\bullet\bullet}Ni'_{Zr}$ configurations, showing the lowest energy structure after DFT relaxation. **d,** Fragments of DFT relaxed hydrated structures with MO_6 and NiO_6 octahedra of $BaZrO_3$ doped with M cations associated with Ni^{3+} with three different types of hydration process. **e,** Hydration energies of free and trapped oxygen vacancies ($M'_{Zr}v_O^{\bullet\bullet}Ni'_{Zr}$) in $BaZrO_3$ doped with M cations, with a schematic illustrating showing three different types of hydration processes.

References

(1) Kreuer, K.-D. Proton-conducting oxides. *Annual Review of Materials Research* **2003**, *33* (1), 333-359. DOI: 10.1146/annurev.matsci.33.022802.091825.

(2) Jones, L. Quantitative ADF STEM: acquisition, analysis and interpretation. *IOP Conference Series: Materials Science and Engineering* **2016**, *109* (1), 012008. DOI: 10.1088/1757-899X/109/1/012008.

(3) Pardoe, D. I.; Simon, L.; Young, D. STAT 501 Regression Methods. Penn State Eberly College of Science: https://online.stat.psu.edu/stat501/ science.psu: 2016.
